# Supplementary material for: MetaRibo-Seq measures translation in microbiomes
Source: Nat Commun. 2020 Jun 29;11:3268. doi: 10.1038/s41467-020-17081-z (PMC7324362; doi:10.1038/s41467-020-17081-z)
Supplement: Supplementary file 10 — Supplementary Data 7 [file 41467_2020_17081_MOESM10_ESM.zip › File2/Confidence_High_Taxonomy/145018_out.krona.html]

Javascript must be enabled to view this page.

members
magnitude
magnitudeUnassigned
count
unassigned
taxon
rank

145018\_out

1

2759
1
superkingdom

kingdom
1
4751

subkingdom
1
451864

4890
phylum
1

1
subphylum
147537

4891
1
class

order
1
4892

family
1
766764

1539665
genus
1


SRS075341\_contig\_number\_15228
268492
species
1
